# Immune microenvironment features underlying the superior efficacy of neoadjuvant immunochemotherapy over chemotherapy in local advanced gastric cancer

**DOI:** 10.3389/fimmu.2025.1497004

**Published:** 2025-01-27

**Authors:** Ning Zhang, Chunyu Li, Zehua Zhao, Biying Jiang, Wentao Wang, Fujing Sun, Yong Zhang, Yanmei Zhu

**Affiliations:** ^1^ Department of Pathology, Affiliated Cancer Hospital of Dalian University of Technology (Liaoning Cancer Hospital and Institute, Cancer Hospital of China Medical University), Shenyang, China; ^2^ Department of Gastric Surgery, Affiliated Cancer Hospital of Dalian University of Technology (Liaoning Cancer Hospital and Institute, Cancer Hospital of China Medical University), Shenyang, China

**Keywords:** gastric cancer, tumor-infiltrating immune cells, tumor immune microenvironment, neoadjuvant therapy, immunochemotherapy

## Abstract

**Background:**

The therapeutic efficacy of neoadjuvant immunotherapy combined with chemotherapy (Io+Chemo) is superior than chemotherapy alone (Chemo). However, the mechanism of Io+Chemo superiority remains to be further elucidated.

**Methods:**

The study included 128 patients with resectable stage II-III gastric cancer, in which 63 were given neoadjuvant Io+Chemo, and 65 Chemo alone. Patients given Io+Chemo were treated with 2-4 cycles of PD-(L)1 inhibitor (Pembrolizumab, Sintililimab or Nivolumab) with S-1 and oxaliplatin (SOX) or capecitabine and oxaliplatin (XELOX) before surgical resection. Patients given Chemo were treated with 2-4 cycles of SOX or XELOX before surgical resection. Tumor tissues were evaluated for tumor-infiltrating immune cells (TIICs) using immunohistochemistry and QuPath software quantitative analysis, for detecting T, B, NK, plasma cells, and macrophages. The relationship between TIICs and different neoadjuvant treatment regimens and pathological responses was also explored.

**Results:**

Compared with Chemo, Io+Chemo induced higher rates of pathological complete response (33.3 vs. 9.2%, p=0.001) and major pathological response (MPR) (49.2 vs. 30.8%, p=0.033). Compared with Chemo group, density of CD4^+^(1904.8 vs. 1530), CD8^+^(1982.9 vs. 1124.4), CD20^+^(1115.6 vs. 574), CD38^+^(1580.4 vs. 1128), CD138^+^(1237.2 vs. 496.4), and CD56^+^ (596.8 vs. 159) cells was increased 24.5%, 76.4%, 94.4%, 40.1%, and 149.2% respectively, whereas CD163^+^ macrophages (994.4 vs. 1706) was decreased 41.7% in Io+Chemo group.

**Conclusions:**

Our study favors neoadjuvant Io+Chemo over Chemo and reveals Io+Chemo can induce the formation of an immune-activated microenvironment that make Io+Chemo superior to Chemo.

## Introduction

Gastric cancer is one of the malignancy that seriously threatens our health, having the fifth highest incidence rate and the fourth highest mortality rate in the world. The incidence of the disease in men is six times higher than in women (1). In recent years, neoadjuvant therapy (NAT) has become increasingly common in local advanced gastric cancer (LAGC). It aims at shrinking the primary tumor, eliminating micrometastases, downstaging tumors and increasing the rate of R0 resection. However, the pathological complete response (pCR) rate of chemotherapy alone (Chemo) is not satisfactory, only 4.6 to 10% (2, 3). Therefore, the development of new treatment approaches to improve the pCR rate of NAT and to prolong patients’ survival is an important clinical challenge.

Currently, the remarkable efficacy of PD-(L)1 inhibitor is receiving increasing attention from researchers in different fields (4). Numerous scientific and clinical studies have demonstrated that PD-(L)1 antibodies produce significant inhibition of tumor cell proliferation through activating immune cells in the tumor immune microenvironment (TIME) in various tumors (5). NAT using PD-(L)1 inhibitors has been shown to be effective in cholangiocarcinoma (6), melanoma (7), lung cancer (8), and colon cancer (9). The pCR rates were about 33.3-50.9% (10, 11), significantly higher than previous conventional treatments. Recent studies on non-small cell lung cancer have shown that compared with chemotherapy alone, neoadjuvant Io+Chemo can significantly improve major pathological response (MPR), pathological complete response (pCR), OS, and event-free survival (EFS) (12). These results are similar to those found in gastric cancer studies.

Immune checkpoint inhibitor therapy has made great strides in the field of treating LAGC patients. Nivolumab combined with chemotherapy has been approved by the FDA for those with advanced and also metastatic gastric cancer (13). Currently, several clinical trials are adding immune checkpoint inhibitors to neoadjuvant treatment regimens for gastric cancer. A study enrolling 749 Asian patients compared the efficacy of nivolumab in combination with chemotherapy to chemotherapy alone in patients with advanced gastroesophageal junction (GEJ)/gastric cancer. It found that patients with the addition of nivolumab generally had longer progression-free survival (PFS), OS, and better overall response rate (14). The other clinical trials including CheckMate 649, KEYNOTE-059, and KEYNOTE-061 all confirmed the superiority of neoadjuvant immunotherapy combined with chemotherapy (Io+Chemo) compared to chemotherapy alone (Chemo) (15).The ORIENT-16 randomized clinical trial confirmed that among patients with previously untreated advanced gastric or GEJ adenocarcinoma, adding sintilimab to chemotherapy significantly improved OS, compared with placebo with chemotherapy (16). However, the superiority of Io+Chemo remains to be further investigated and the associated mechanisms elucidated.

The TIME includes immune cells, cancer-associated fibroblasts, vascular endothelial cells, etc. (17). By cross-talking with cancer cells, it plays a crucial role in the multiple biological behavior of cancer, including an impact on the efficacy of NAT. PD-(L)1 inhibition enhances the initiation of T cells in the TIME by utilizing large amount of tumor antigens in the primary tumor and also restores the function of tumor-specific cytotoxic T cells (18). Therefore, to illustrate the mechanisms underlying the superior efficacy of Io+Chemo, it is essential to analyze the tumor-infiltrating immune cells (TIICs) in tumor specimens obtained after NAT.

In this study, we retrospectively collected 128 surgical specimens of LAGC patients who had received NAT, including 63 cases of neoadjuvant Io+Chemo and 65 cases of neoadjuvant Chemo. We also explored the relationship of TIICs with treatment modality and pathological response in an attempt to uncover the mechanisms responsible for the superior efficacy of neoadjuvant Io+Chemo.

## Materials and methods

### Research design

**Figure 1:**
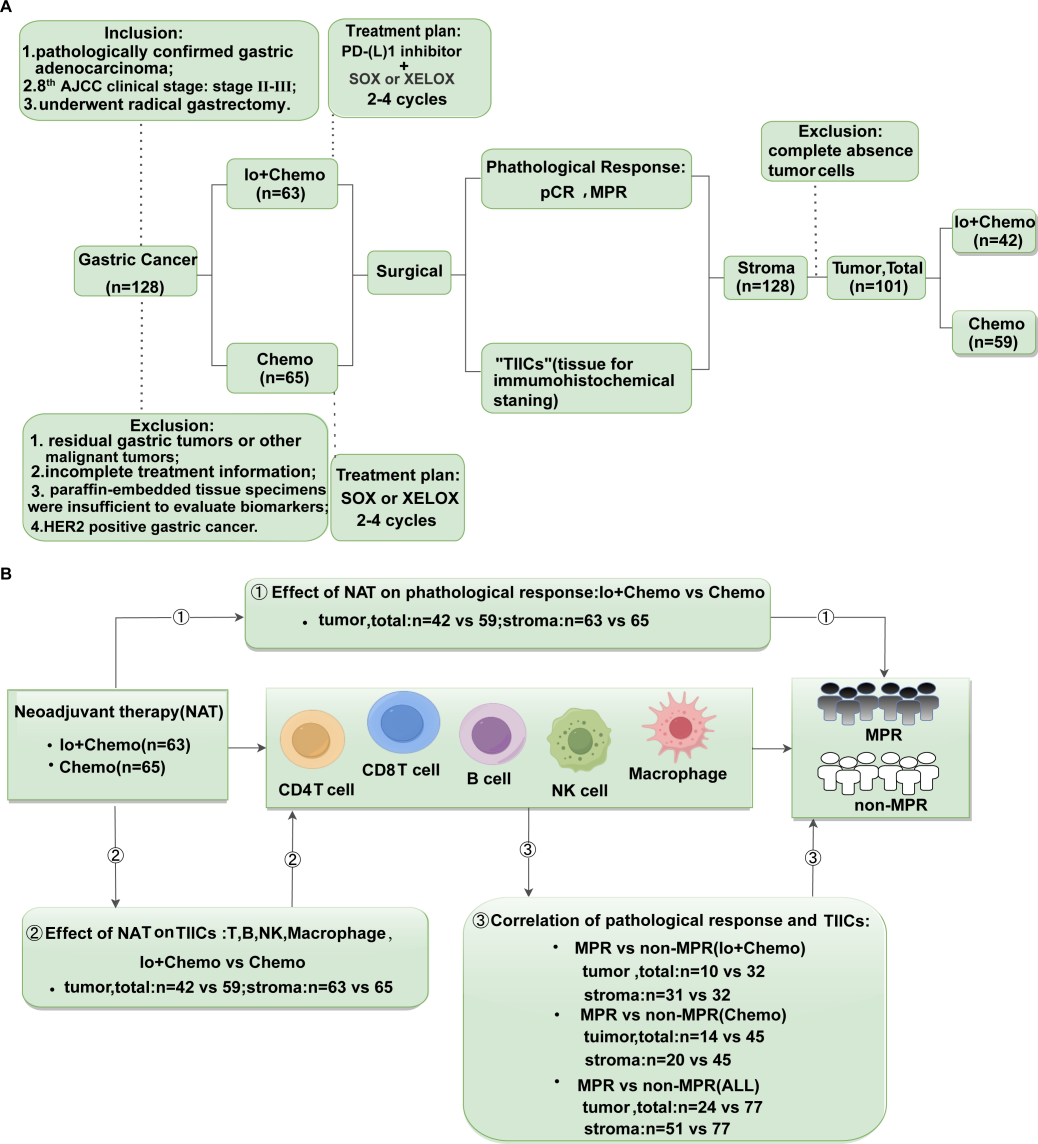
Study design examining effects of neoadjuvant therapies on resectable LAGC patients. **(A)** Study flow chart depicting the study protocol. According to our inclusion and exclusion criteria, a total of 128 patients with gastric cancer who underwent surgical resection after neoadjuvant therapy were included in the trial, of which 63 received Io+Chemo and 65 received Chemo. Their pathological response and TIICs of tumor, stromal, and total (tumor + stroma) were evaluated. All samples have been evaluated TIICs in stroma. Due to 27 patients achieved pCR, i.e., no tumor cells remained, TIICs were only evaluated in stroma of these samples. **(B)** The endpoints explored and sample details in each analysis. ① Evaluated the pathological response of patients received neoadjuvant Io+Chemo and Chemo; ② Evaluate the effect of neoadjuvant Io+Chemo and Chemo on TIICs; ③ Evaluated the relationship between pathological response and TIICs in different treatment subgroups. LAGC, local advanced gastric cancer; TIICs, Tumor infiltrating immune cells; NAT, neoadjuvant therapy; pCR, pathological complete response; MPR, major pathological response; Io+Chemo, immunochemotherapy; Chemo, chemotherapy.

LAGC patients given NAT (Io+Chemo or Chemo) at Liaoning Cancer Hospital from 2019 to 2022 were retrospectively included. Patients were selected for the study according to inclusion criteria: (1) pathological verification of gastric adenocarcinoma, for adenocarcinoma is the most common histological type; (2) AJCC clinical stage: Stage II-III, for patients in stage I can undergo direct surgical resection without neoadjuvant therapy, patients in stage IV are treated with conversion therapy rather than neoadjuvant therapy; (3) underwent radical gastrectomy. The exclusion criteria were the following: (1) existence of residual gastric tumors or other malignant tumors; (2) the treatment information was incomplete; (3) paraffin-embedded tissue specimens were insufficient to evaluate biomarkers; and (4) HER2 positive gastric cancer, for these patients would receive HER2-targeted therapy instead of Io+Chemo. Patients with Io+Chemo were treated with 2-4 cycles of PD-(L)1 inhibitor (Pembrolizumab, Sintililimab or Nivolumab) with SOX or XELOX before surgical resection, and patients with Chemo were treated with 2-4 cycles of SOX or XELOX before surgical resection. Samples from surgical resections after neoadjuvant therapy were obtained and evaluated for pathological response and TIICs. Details of the research process are shown in **Figure 1**. All research processes were in full compliance with the Helsinki Declaration (revised in 2013). Approval of our investigation was granted by the Ethics Committee of Liaoning Cancer Hospital and Institute, and patients provided signed informed consent.

### Pathological assessment

Surgically resected specimens were subjected to hematoxylin-eosin (H&E) staining to observe the pathological response after NAT. The grading criteria was based on the Mandard tumor regression grade (TRG) system. TRG1: the tumor completely regressed; TRG2: a small number of residual cancer cells (<10%) dispersed in degenerated fibrous tissues; TRG3: a large number of residual cancer cells, but still dominated by fibrous tissue; TRG4: the proportion of residual cancer cells exceeds that of fibrous degenerated tissue; TRG5: no significant regression of cancer cells. pCR was defined as no residual surviving tumor cells, MPR referred to less than 10% residual tumor cells (19). TRG1 was equal to pCR, TRG1 and TRG2 was equal to MPR.

### Immunohistochemical staining

Immunohistochemical staining was performed to analyze various TIICs. Primary antibodies included CD3 (GA045207, Genentech), CD4 (GT219107, Genentech), CD8 (GT211207, Genentech), CD20 (GM075507, Genentech, ready-to-use), CD38 (GT212907, Genentech), CD138 (GT245107, Genentech), CD56 (GT200507, Genentech), CD68 (GT087607, Genentech), CD163 (GT207707, Genentech). Non-specific blocking agents were used to inhibit endogenous peroxidase activity. Horseradish peroxidase (HRP)-labeled sheep anti-mouse/rabbit immunoglobulin (IgG) polymers were used as secondary antibodies and incubated with their respective antibodies. The slides were stained with 3.3-diaminobenzidine (DAB) and re-stained with hematoxylin. All slides were scanned and digitized with a high-throughput scanner (Nanozoomer S360, Hamamatsu).

### TIIC quantitative analysis by QuPath software

**Figure 2:**
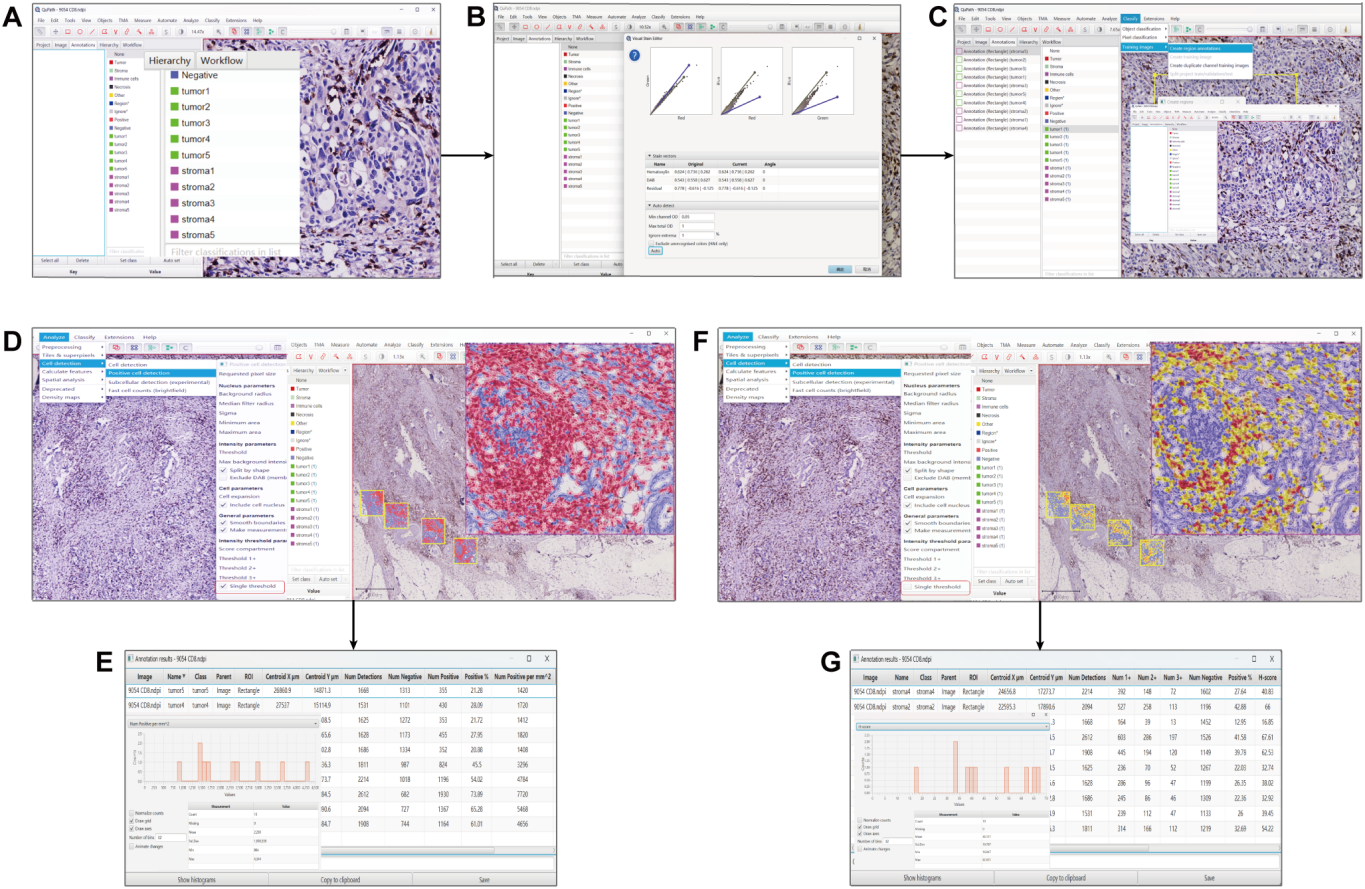
Import scanned slides into QuPath software and **(A)** Create annotations for tumor and stroma. **(B)** Separating stains, check “Estimate stain vectors” for better cell detection. **(C)** Select five suitable sections in each of the tumor and stroma, each with an area of 500 × 500 μm, and add annotations for them. **(D)** Select "Analyze" →"cell detection"→"positive detection"→check "Single threshold". **(E)** Select "show annotation measurements" to export the data which contains percentage and density. **(F)** Select "Analyze" →"cell detection"→"positive detection"→uncheck "Single threshold". **(G)** Select "show annotation measurements" to export the data which contains H-score values.

QuPath is novel open-source software that can be used for quantitative analysis. The source code is available at https://qupath.github.io. The scanned digital images were imported into the QuPath software for data analysis. The operation flow of QuPath is shown in **Figure 2**. Step 1. Setting the image type. In our study, we chose the image type Brightfield H-DAB and created annotations for the tissue regions to be analyzed in the analysis panel; Step 2. Run Estimate stain vectors to separate stains for better cell detection; Step 3: Select five regions of interest in tumor interior and stroma manually selected by a pathologist with diagnostic experience, and fix the format size of each region as 500×500μm (20) to ensure that the measurement area is consistent and add the annotations that have been created; Step 4: Positive cell density, positive cell percentage and H-score value were analyzed and counted for the tumor interior, tumor stroma and tumor as a whole, i.e., tumor interior plus tumor stroma. The positive cell density refers to the number of positive cells per unit area. The positive cell percentage refers to the proportion of positive cells among all cells per unit area. H-score means the product of positive cell percentage and positive intensity per unit area.

### SPSS statistical analysis

Statistical analysis was performed using SPSS Statistics 25 software. The Pearson’s chi-square test was used to analyze the relationship between the two treatment modalities and clinicopathological factors. If the continuous variables were normally distributed, the unpaired t test was used to compare the variables; otherwise, the Mann-Whitney U test was used. Survival analysis was performed and plotted using Kaplan-Meier method, and the log-rank test was used to compare differences. P < 0.05 was considered statistically significant.

## Results

### Baseline characteristics

Between 2019 and 2022, 128 patients participated in our study; their ages were from 25 to 85 years old, showing a median of 60, and there were 90 men and 38 women. All patients received either NAT Io+Chemo or Chemo, of which 63 patients received Io+Chemo and 65 patients received Chemo. Baseline characteristics were balanced between NAT Io+Chemo and Chemo. The clinicopathological features of all patients are shown in **Table 1**. Compared to the Chemo group, the ypT staging (p=0.002), ypN grading (p=0.01) and ypTNM staging (p=0.005) of tumors were significantly lower in the Io+Chemo group.

**Table 1:** Baseline characteristics of LAGC patients with neoadjuvant therapy.

	All	Chemo	Io+Chemo	P value
Age (yr)				0.251
Median (range)	59.71 (25~85)	60.06 (25~79)	59.35 (33~85)	
<55	81 (63.3)	38 (58.5)	43 (68.3)	
≥55	47 (36.7)	27 (41.5)	20 (31.7)	
Gender				0.152
Male	90 (70.3)	42 (64.6)	48 (76.2)	
Female	38 (29.7)	23 (35.4)	15 (23.8)	
cTNM, before NAT				0.288
II	43 (33.6)	19 (29.2)	24 (38.1)	
III	85 (66.4)	46 (70.8)	39 (61.9)	
ypT				**0.002**
0-2	44 (34.4)	14 (21.5)	30 (47.6)	
3-4	84 (65.6)	51 (78.5)	33 (52.4)	
ypN				**0.010**
0-1	77 (60.2)	32 (49.2)	45 (71.4)	
2-3	51 (39.8)	33 (50.8)	18 (28.6)	
ypTNM				**0.005**
1	40 (31.3)	13 (20)	27 (42.9)	
2-3	88(68.8)	52(80)	36(57.1)	
Histological type				0.058
Adenocarcinoma	94(73.4)	43(66.2)	51(81.0)	
Poorly cohesive carcinoma	34(26.6)	22(33.8)	12(19.0)	
Grade of differentiation				0.072
Well and moderately differentiated	45(35.2)	18(27.7)	27(42.9)	
Poorly differentiated	83(64.8)	47(72.3)	36(57.1)	
Lauren classification				0.052
Intestinal	52(40.6)	21(32.3)	31(49.2)	
Diffuse or mixed	76(59.4)	44(67.7)	32(50.8)	
Nervous invasion				0.606
No	58(45.3)	28(43.1)	30(47.6)	
Yes	70(54.7)	37(56.9)	33(52.4)	
Vascular or lymphatic invasion				0.379
No	66(51.6)	36(55.4)	30(47.6)	
Yes	62(48.4)	29(44.6)	33(52.4)	
PD-L1				0.613
CPS<5	76(59.4)	40(61.5)	36(57.1)	
CPS≥5	52(40.6)	25(38.5)	27(42.9)	
MMR				0.848
pMMR	111(86.7)	56(86.2)	55(87.3)	
dMMR	17(13.3)	9(13.8)	8(12.7)	

LAGC, local advantage gastric cancer. Bold values were statistically significant (P < 0.05).

### Adding immunotherapy improves NAT efficacy

After NAT, the pathological response of each patient's primary tumor was evaluated. A total of 27 patients out of 128 patients achieved pCR, and 51 patients achieved MPR. There was a significant increase in pCR in the Io+Chemo group (33.3 vs 9.2%, p=0.01), and the MPR rate was numerically increased (49.2 vs 30.8%, p=0.033) compared with Chemo. We also compared the OS of the two groups, the results showed that the OS of patients recieved Io+Chemo was longer than that of the patients recieved Chemo alone. However, there was no statistically significant (p=0.449) (**Supplementary Figure S1**).

### Comparison of TIIC subsets in patients treated with neoadjuvant Io+Chemo versus Chemo

**Figure 3:**
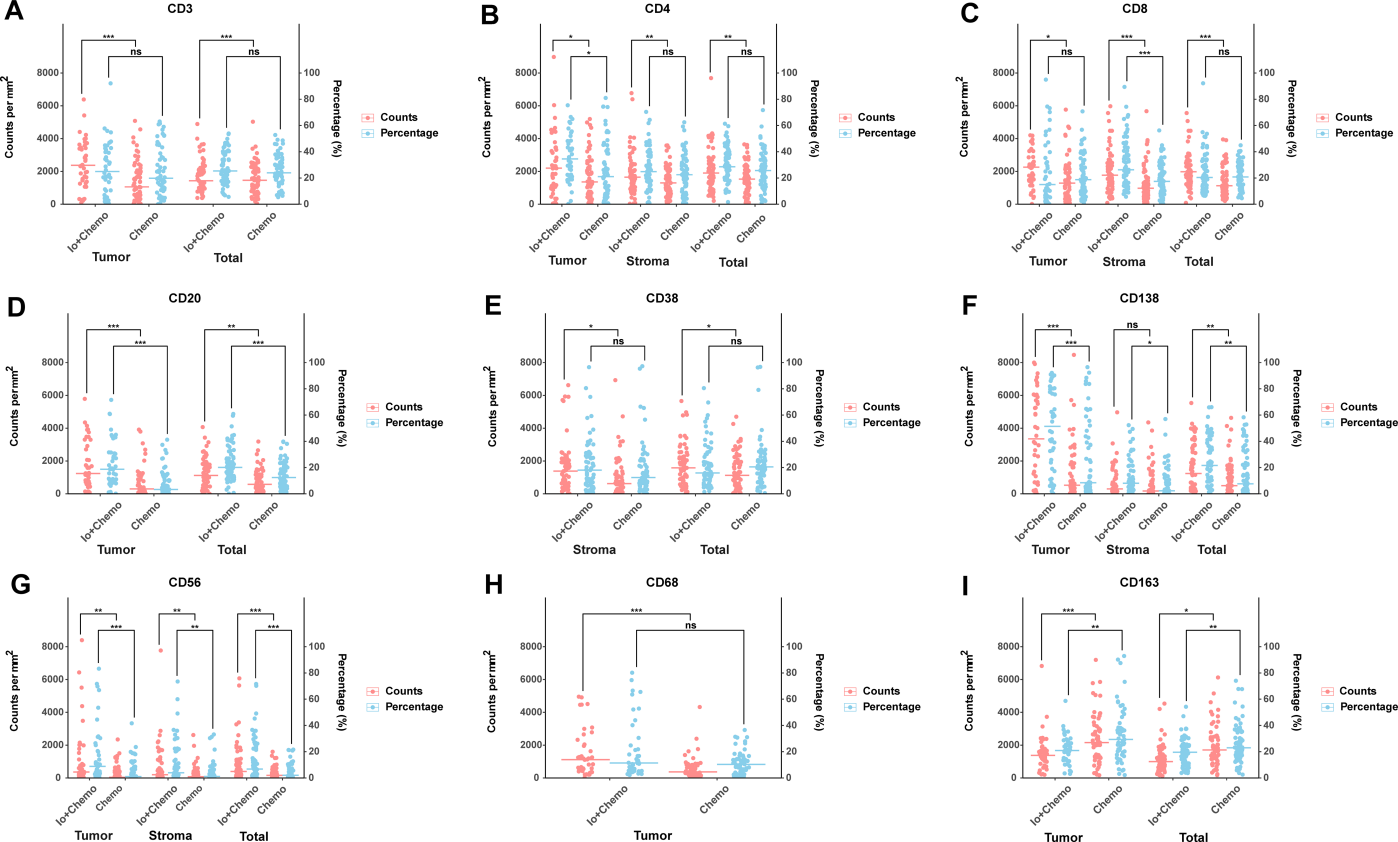
Comparison of TIIC subsets in patients treated with neoadjuvant Io+Chemo versus Chemo. The scatter plot was shown as median. The density and percentage of CD3 **(A)**, CD4 **(B)**, CD8 **(C)**, CD20 **(D)**, CD38 **(E)**, CD138 **(F)**, CD56 **(G)**, CD68 **(H)**, CD163 **(I)** were statistical different in responders and non-responders. Figure was created with R. *p*<*0.05; **p*<*0.01; ***p<0.001; ns, no statistical significance.

**Figure 4:**
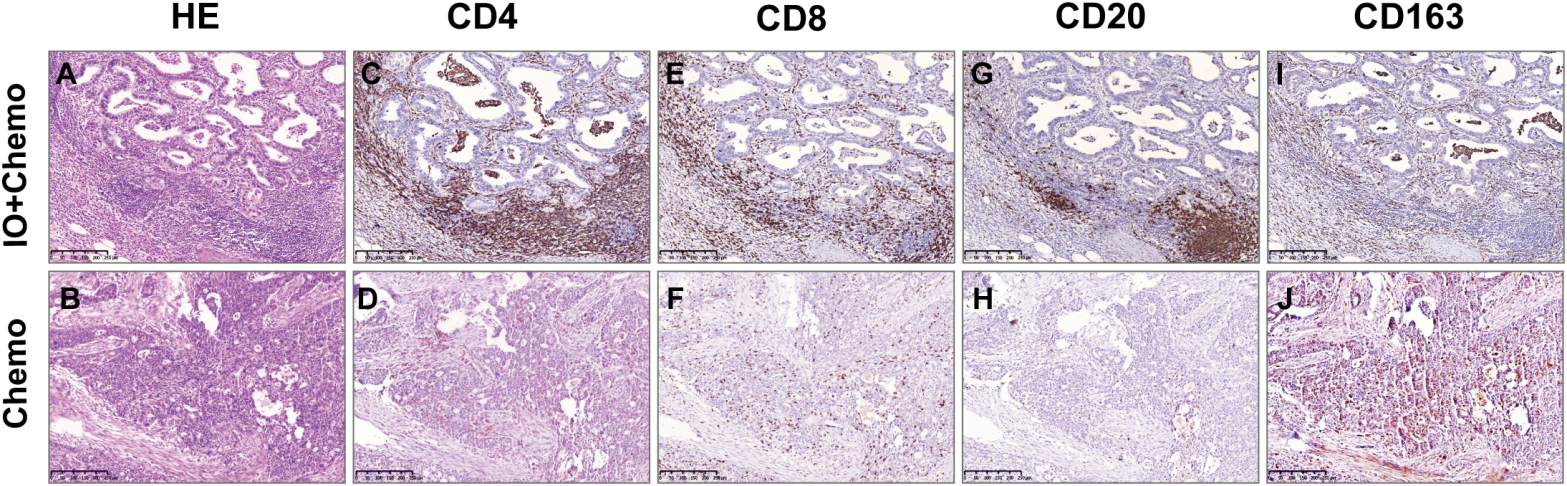
Comparison of TIIC between Io+Chemo and Chemo by immunohistochemistry. HE: the more immune cells in Io+Chemo specimens **(A)** than in Chemo specimens **(B)**. CD4: the density of Io+Chemo specimens **(C)** was higher than Chemo specimens **(D)**. CD8: the density of Io+Chemo specimens **(E)** was higher than Chemo specimens **(F)** CD20: the density of Io+Chemo specimens **(G)** was higher than Chemo specimens **(H)**. CD163: the density of Io+Chemo specimens **(I)** was lower than Chemo specimens **(J)**.

Surgically resected tissue samples were subjected to immunohistochemical staining to observe TIICs after NAT. No tumor cell residues were seen in 27 of the 128 tissue samples. Therefore, TIICs were evaluated in 128 stroma and 101 tumor samples. The details of TIIC subsets in patients treated with neoadjuvant Io+Chemo versus Chemo are shown in **Figures 3**, **4** and **Supplementary Table S1**. The CD3^+^ cells showed a significantly higher density in tumor and total as well in the Io+Chemo group than in Chemo (tumor, p=0.000; total, p=0.000). Compared with the Chemo group, the Io+Chemo group exhibited greater infiltration by CD4^+^ cells in tumor, stroma and total (density: p= 0.022; stroma, p=0.006; total, p=0.010; percentage: tumor, p=0.020; H-score: tumor, p=0.010). The CD8^+^ cells were significantly more abundant in tumor, stroma and total in the Io+Chemo group versus Chemo alone (density: tumor, p=0.017; stroma, p=0.000; total, p=0.000; percentage: stroma, p=0.001; H-score: tumor, p=0.016). There was a higher degree of infiltration of CD20^+^ cells in tumor and total as well in the Io+Chemo group than that in Chemo alone (density: tumor, p=0.000, total, p=0.002; percentage: tumor, p=0.000, total, p=0.000; H-score: tumor, p=0.000). In contrast to the Chemo group, the Io+Chemo group had a significantly greater density of CD38^+^ cells in both stroma and total (stroma, p=0.011, total, p=0.039). The CD138^+^ cells showed a higher density, percentage and H-score in tumor, stroma and total in the Io+Chemo group compared with Chemo (density: tumor, p=0.000, total, p=0.002; percentage: tumor, p=0.000, stroma, p=0.022, total, p=0.003; H-score: tumor, p=0.000, stroma, p=0.032, total, p=0.000). The CD56^+^ cells were significantly more abundant in tumor, stroma and total in the Io+Chemo group compared with Chemo (density: tumor, p=0.002, stroma, p=0.007, total, p=0.000; percentage: tumor, p=0.000, stroma, p=0.009, total, p=0.000; H-score: tumor, p=0.001, stroma, p=0.000, total, p=0.000). Compared with the Chemo group, there was a higher density of CD68^+^ cells in tumor in the Io+Chemo group (p=0.000). A lower degree of infiltration of CD163^+^ macrophages occurred in both tumor and total in the Io+Chemo versus Chemo (density: tumor, p=0.001, total, p=0.018; percentage: tumor, p=0.003, total, p=0.004; H-score: tumor, p=0.000, total, p=0.007).

### TIIC subsets in MPR versus non-MPR patients in neoadjuvant Io+Chemo group

Similarly, in the neoadjuvant Io+Chemo group, a total of 31 patients achieved MPR and 32 patients were non-MPR. We compared the TIICs between different treatment effects in this group. The details of TIIC subsets difference are shown in **Supplementary Table S3**. CD3^+^ cells exhibited a higher degree of ifiltration in both tumor, stroma and total in the MPR versus non-MPR group (density: stroma, p=0.001; percentage: tumor, p=0.038). A higher percentage of CD8^+^ cells was observed in the MPR group compared with non-MPR (tumor, p=0.001, stroma p=0.034). CD20^+^ cells displayed a significantly higher abundance in percentage in stroma in the MPR than non-MPR (p=0.032). CD138^+^ cells showed a higher degree of infiltration in tumor, stroma and total in MPR over non-MPR (density: tumor, p=0.043, total, p=0.000; percentage: total, p=0.023; H-score: stroma, p=0.011, total, p=0.021). There was a significantly higher amount of CD68^+^ cells in the MPR than non-MPR group (density: stroma, p=0.002; percentage: stroma, p=0.002; H-score, stroma, p=0.006). A lower percentage of CD163^+^ cells was observed in tumor, stroma and total in MPR than non-MPR (percentage: tumor, p=0.041, stroma, p=0.001, total, p=0.000).

### TIIC subsets in MPR versus non-MPR patients in neoadjuvant Chemo group

In the neoadjuvant Chemo group, a total of 20 patients achieved MPR and 45 patients were non-MPR. We compared the TIICs in MPR versus non-MPR patients in this group. The details of TIIC subsets difference are shown in **Supplementary Table S4**. The CD3^+^ cells had a higher H-score in the MPR than non-MPR group (total, p=0.019). Compared with the non-MPR group, the MPR group exhibited greater infiltration by CD4^+^ cells in stroma and total (density: stroma, p=0.017, total, p=0.002; percentage: total, p=0.018; H-score: total, p=0.037). A significantly higher H-score of CD20^+^ cells was observed in MPR versus non-MPR (stroma, p=0.022, total, p=0.022). A higher infiltration degree of the CD38^+^ cells was seen in stroma in the MPR than non-MPR group (density, p=0.023; H-score, p=0.030). The density of CD56^+^ cells was greater in the MPR than non-MPR group (stroma, p=0.009). The infiltration by CD163^+^macrophages was significantly less in MPR versus non-MPR (density: stroma, p=0.000, total, p=0.001; percentage: stroma, p=0.002, total, p=0.003; H-score: stroma, p=0.012, total, p=0.038).

All patients, including both in neoadjuvant Io+Chemo group and in neoadjuvant Chemo group, were grouped into MPR and non-MPR. The difference of TIICs subsets between MPR and non-MPR are shown in **Supplementary Table S2** and **Supplementary Figure S2**. In addition, we compared the TIICs between pCR and non-pCR groups in stroma regardless of treatment regimen. Compared with the non-pCR group, the pCR group exhibited greater infiltration by CD20^+^ cells (percentage: p=0.024), CD3^+^ cells (density: p=0.010), and CD138^+^ cells (H-score: p=0.001) in stroma. A lower degree of infiltration of CD163^+^ macrophages occurred in stroma in the pCR versus non-pCR (density: p=0.000; percentage: p=0.000; H-score: p=0.000).

## Discussion

In this study, compared with neoadjuvant Chemo alone, Io+Chemo achieved better pCR and MPR rates. Quantitative TIICs analysis showed a higher degree of TIIC infiltration except CD163^+^ macrophages in the population receiving Io+Chemo, in the MPR subgroup of Io+Chemo patients, in the MPR subgroup of Chemo patients, and in the MPR group of all patients.

Our results suggests that the neoadjuvant Io+Chemo rather than Chemo alone is more beneficial to patients with LAGC. Previous studies have shown that the mean pCR rate of LAGC patients treated with neoadjuvant Chemo alone is about 6.7% (2, 3), and that the average pCR rate for LAGC patients receiving neoadjuvant Io+Chemo can range from 19.4 to 33.6% (21–24). The recent study of the NEOSUMMIT-01 clinical trial showed that the addition of PD-1 monoclonal antibody to perioperative chemotherapy in patients with cT3-4aN+M0 resectable gastric or GEJ adenocarcinoma significantly increased the pCR rate compared with the SOX/XELOX chemotherapy group alone (22.2% vs. 7.4%) (25). The RATIONALE-305 trial confirmed that in patients with HER2-negative advanced gastric cancer or GEJ adenocarcinoma, tislelizumab combined with chemotherapy significantly improved OS compared with chemotherapy alone (26). The data are similar to ours. Similar findings have been seen in studies of other cancer types. The BGB-A317-2002 study showed that the pCR rate of patients who underwent radical surgery for neoadjuvant treatment of muscle-invasive bladder cancer with tislelizumab combined with gemcitabine/cisplatin reached 50.9%. Updated follow-up data showed that the 1-year EFS, OS, and RFS rates were 89.3%, 91.2%, and 85.2% respectively (11). The result indicated the positive clinical significance of Io+Chemo. CheckMate 816 reported that neoadjuvant nivolumab along with chemotherapy induced significantly higher pathological responses than did chemotherapy alone in stage IB to IIIA resectable non-small cell lung cancer (NSCLC) (26). Similarly, multiple clinical trials showed that PD-(L)1 blockade plus Chemo significantly increased MPR and pCR rates in NSCLC (27–30).Mechanistically, chemotherapeutic agents are capable of both direct killing of tumor cells and indirect inhibition of tumor development by promoting the release of tumor antigens to regulate tumor TIME. Moreover, authors have demonstrated that PD-L1 expression on cancer cells can be induced by chemotherapy. So immunotherapy plus chemotherapy can synergistically improve the therapeutic efficacy of anti-PD-(L)1 monotherapy (31, 32). Combination strategies are becoming "major players" in neoadjuvant treatment regimens of LAGC.

Although the molecular mechanisms of PD-(L)1 inhibition in cancer treatment have been investigated, the mechanisms by which Io+Chemo is superior to Chemo alone are not well elucidated. QuPath software, an open-source solution for whole slide image analysis and digital pathology (33), was used to quantitatively assess the TIICs in the TIME in our study. Accordingly, immunohistochemically stained positive cells can be quantified, and the percentage of positive cells and the H-score value per unit area and have been widely used in the field of quantitative analysis of digital pathology. In our study, more CD8^+^ T cells infiltrated in the Io+Chemo group compared to the Chemo group. Tang et al. established four groups of tumor-bearing mouse models: control (with PBS), anti-PD-1 antibody treatment, chemotherapy, and chemotherapy with PD-1 blockade. Compared with chemotherapy alone, chemotherapy with PD-1 blockade caused a significant increase in infiltration degree of CD8^+^ effector T cells (34). Similar evidence has been reported by other research groups in a variety of tumor types, including esophageal squamous cell carcinoma (35),ovarian cancer (36),and lung cancer (8, 37). Some researchers have reported that large numbers of CD8^+^ T cells within or around tumors are associated with improved disease-free survival and OS (38). In tumors, cancer cells inhibit the activation of CD8^+^ T cells (cytotoxic T lymphocytes, CTL) by binding inhibitory checkpoints on the CTL surface such as PD-L1, which is believed to be a critical form of cancer cell immune escape (39). Immune checkpoint inhibitors can competitively bind to PD-1 on the surface of cancer cells to activate the CTL. In the "activated" state, the CTL recognizes MHC-I-like molecules on the cancer cell surface and kills the target cells via granule cytosolization (granzyme A and B) and necrosis and apoptosis triggered by death ligands in the presence of chemokines. In addition, CTL also can induce cytotoxic effects on cancer cells by secreting interferon-γ and tumor necrosis factor α (40). In our study, CD4^+^ T cell number was higher in Io+Chemo compared to Chemo. CD4^+^ T cells (follicular helper T cells), are one of the most abundant and essential effector T cells (41). Many studies have shown that CD4^+^ helper T cells participate in anti-tumor immune responses and play a key role in determining tumor responsiveness to immune checkpoint blockade immunotherapy (42). Under certain conditions, CD4^+^ T cells can possess cytotoxic activity and secrete cytotoxic particles containing perforins and granzymes that directly kill target cells. These cells seem to be derived from Treg cells, not effector CD4^+^ T cells (43). They can also promote CTL proliferation and activation and memory CTL formation (44). These mechanisms explain why Io+Chemo is superior to Chemo to some extent.

In our study, CD20^+^ and CD138^+^ cells infiltrated more in Io+Chemo than Chemo group. The same result was found by Tang et al. (34). Mature B cells are able to deliver tumor-associated antigens directly to T cells for tumor cell kill. Antibodies actively secreted by these B cells recognize tumor antigens to effectively control tumor progression (45). There is a negative correlation of B cell number in the tumor with lung cancer stage (46). Besides T and B cells, CD56^+^ NK cells infiltrated more in Io+Chemo group. NK cells are an important subpopulation of immune cells that antagonize tumors. NK cells have a similar function as CD8^+^ T cells in TIME (47), inducing apoptosis of target cells mainly through the release of perforin and granzyme (48). They also promote cross-presentation of antigens to CTLs and influence the anti-tumor response of T cells (47). After migrating to cancer nests, macrophages become tumor-associated macrophages (TAMs). Authors have reported that there are significantly more infiltrating TAMs in GC tissues than in adjacent tissues, indicating that with the development of GC, there is recruitment of TAMs and close cross-talk with cancer cells (49). On one hand, TAMs exhibit antitumor effects via the phagocytosis and killing of cancer cells; on the other hand, after interaction with cancer cells, TAM may change phenotype, i.e., M1 macrophages are converted to M2 macrophages. M1 macrophages promote inflammatory responses and usually have antitumor effects; M2 macrophages contribute to immune escape, neovascularization and remodeling of the extracellular matrix, which promote tumor development (50). In our study, CD68^+^ TAM infiltrated to a higher extent, while lower numbers of CD163^+^ M2 macrophages infiltrated in the Io+Chemo group. Tang et al. found that the M1/M2 macrophage ratio was significantly higher in mice treated with neoadjuvant Io+Chemo compared to Chemo alone (34). These findings indicate that immunotherapy can promote the infiltration of CD4^+^cells, CD8^+^ T cells, B cells and NK cells, and reduce the number of M2 macrophages, which in turn reduces immunosuppression and exerts anti-tumor effects.

We further investigated the relationship between pathological response and TIICs in different treatment subgroups. In the Io+Chemo group, patients with MPR had significantly higher CD3^+^, CD4^+^, CD8^+^, CD20^+^, CD38^+^, CD138^+^, and CD68^+^ cell infiltration and significantly lower CD163^+^ macrophage infiltration. In samples of cTNM-III gastric cancer patients who received neoadjuvant Io+Chemo and surgery, Tang et al. found that a greater proportion of CD8^+^ T cells, M1/M2 macrophages and plasma cells in the responders' than non-responders’ TIME (34). Wei et al. also found an increase in the number of CD8^+^ T cells and the ratio of M1/M2 in patients with GC who benefited from NAC plus PD-1 blockade (51). In addition, mIF analysis showed that capecitabine + oxaliplatin + pembrolizumab (COP ) increased the densities of CD3^+^ and CD8^+^ T cells in G/GJ adenocarcinomas, and the degree of pathological reaction was related to the increase of aggregation of PanCK^+^ cells to CD3^+^ cells (52).The results above are similar to ours. In patients received chemotherapy alone, CD3^+^, CD4^+^, CD20^+^, CD38^+^, and CD56^+^ cell infiltration was significantly higher in MPR patients, and the results for CD163^+^ macrophages were similar to those in the Io+Chemo group. Xing et al. Found that the levels of CD8^+^ T cells, CD20^+^ B cells, and CD57^+^ NK cells significantly increased in MPR group. Univariate analysis showed that the levels of CD20^+^ B cells, CD68^+^ macrophages, and CD8^+^ T cells were correlated with a good response (53). All of these results suggested that TIIC infiltration in TIME is the reason for prominent pathological response regardless of treatment strategy.

The main limitation of our investigation was that it was a retrospective design. In the future, we will conduct prospective studies to compare the impact of different neoadjuvant treatment methods on the TIME of gastric cancer. The second limitation was that the absence of pre-treatment baseline data on immune cell infiltration limited the context for post-treatment comparisons. In the future, we intend to design a study that includes pre-treatment biopsy specimens to compare changes in TIME before and after treatment and to explore TIME biomarkers that can predict efficacy. The third limitation was the lack of longitudinal follow-up data. In the future, we will continue to improve the follow up data.

## Conclusions

Our study favors neoadjuvant Io+Chemo over Chemo and reveals the tumor immune microenvironment characteristics that make Io+Chemo superior to Chemo. Our findings provide new insights into the mechanisms by which Io+Chemo is more efficient than Chemo alone.

## Data Availability

The original contributions presented in the study are included in the article/**Supplementary Material.** Further inquiries can be directed to the corresponding authors.

## References

[1] Sung H Ferlay J Siegel RL Laversanne M Soerjomataram I Jemal A. Global cancer statistics 2020: globocan estimates of incidence and mortality worldwide for 36 cancers in 185 countries. CA: Cancer J Clin. (2021) 71:209–49. doi: 10.3322/caac.21660 33538338

[2] Schneider BJ Shah MA Klute K Ocean A Popa E Altorki N. Phase I study of epigenetic priming with azacitidine prior to standard neoadjuvant chemotherapy for patients with resectable gastric and esophageal adenocarcinoma: evidence of tumor hypomethylation as an indicator of major histopathologic response. Clin Cancer Research: Off J Am Assoc Cancer Res. (2017) 23:2673–80. doi: 10.1158/1078-0432.CCR-16-1896 PMC5425331 27836862

[3] Pereira MA Ramos MFKP Dias AR Cardili L Ribeiro RRE Charruf AZ. Lymph node regression after neoadjuvant chemotherapy: A predictor of survival in gastric cancer. J Surg Oncol. (2020) 121:795–803. doi: 10.1002/jso.25785 31773740

[4] Topalian SL Taube JM Pardoll DM. Neoadjuvant checkpoint blockade for cancer immunotherapy. Sci (New York NY). (2020) 367:eaax0182. doi: 10.1126/science.aax0182 PMC7789854 32001626

[5] Zhang H Liu L Liu J Dang P Hu S Yuan W. Roles of tumor-associated macrophages in anti-pd-1/pd-L1 immunotherapy for solid cancers. Mol Cancer. (2023) 22:58. doi: 10.1186/s12943-023-01725-x 36941614 PMC10029244

[6] Zhang Z Wang D Yang L Zhao L Yang L Zhang J. Efficacy and safety analysis of chemotherapy combined with immunotherapy compared with standard chemotherapy for advanced biliary tract Malignant tumors. Cancer Med. (2023) 12:15217–28. doi: 10.1002/cam4.6209 PMC10417155 37392168

[7] Blank CU Rozeman EA Fanchi LF Sikorska K van de Wiel B Kvistborg P. Neoadjuvant versus adjuvant ipilimumab plus nivolumab in macroscopic stage iii melanoma. Nat Med. (2018) 24:1655–61. doi: 10.1038/s41591-018-0198-0 30297911

[8] Forde PM Chaft JE Pardoll DM. Neoadjuvant pd-1 blockade in resectable lung cancer. New Engl J Med. (2018) 379:e14. doi: 10.1056/NEJMc1808251 30157404

[9] Chalabi M Fanchi LF Dijkstra KK Van den Berg JG Aalbers AG Sikorska K. Neoadjuvant immunotherapy leads to pathological responses in mmr-proficient and mmr-deficient early-stage colon cancers. Nat Med. (2020) 26:566–76. doi: 10.1038/s41591-020-0805-8 32251400

[10] Cai W Jing M Gu Y Bei T Zhao X Chen S. Tumor microenvironment features decipher the outperformance of neoadjuvant immunochemotherapy over chemotherapy in resectable non-small cell lung cancer. Front Immunol. (2022) 13:984666. doi: 10.3389/fimmu.2022.984666 36275670 PMC9582151

[11] Li K Zhong W Fan J Wang S Yu D Xu T. Neoadjuvant gemcitabine-cisplatin plus tislelizumab in persons with resectable muscle-invasive bladder cancer: A multicenter, single-arm, phase 2 trial. Nat Cancer. (2024) 5:1465–78. doi: 10.1038/s43018-024-00822-0 39256488

[12] Sorin M Prosty C Ghaleb L Nie K Katergi K Shahzad MH. Neoadjuvant chemoimmunotherapy for nsclc: A systematic review and meta-analysis. JAMA Oncol. (2024) 10:621–33. doi: 10.1001/jamaoncol.2024.0057 PMC10958389 38512301

[13] Hindson J. Nivolumab plus chemotherapy for advanced gastric cancer and oesophageal adenocarcinoma. Nat Rev Gastroenterol Hepatol. (2021) 18:523. doi: 10.1038/s41575-021-00484-8 34158606

[14] Lin Y Liang H-W Liu Y Pan X-B. Nivolumab adjuvant therapy for esophageal cancer: A review based on subgroup analysis of checkmate 577 trial. Front Immunol. (2023) 14:1264912. doi: 10.3389/fimmu.2023.1264912 37860010 PMC10582756

[15] Fuchs CS Doi T Jang RW Muro K Satoh T MaChado M. Safety and efficacy of pembrolizumab monotherapy in patients with previously treated advanced gastric and gastroesophageal junction cancer: phase 2 clinical keynote-059 trial. JAMA Oncol. (2018) 4:e180013. doi: 10.1001/jamaoncol.2018.0013 29543932 PMC5885175

[16] Xu J Jiang H Pan Y Gu K Cang S Han L. Sintilimab plus chemotherapy for unresectable gastric or gastroesophageal junction cancer: the orient-16 randomized clinical trial. JAMA. (2023) 330:2064–74. doi: 10.1001/jama.2023.19918 PMC10698618 38051328

[17] Oya Y Hayakawa Y Koike K. Tumor microenvironment in gastric cancers. Cancer Sci. (2020) 111:2696–707. doi: 10.1111/cas.14521 PMC7419059 32519436

[18] Bretz AC Parnitzke U Kronthaler K Dreker T Bartz R Hermann F. Domatinostat favors the immunotherapy response by modulating the tumor immune microenvironment (Time). J Immunotherapy Cancer. (2019) 7:294. doi: 10.1186/s40425-019-0745-3 PMC6839078 31703604

[19] Rojas F Parra ER Wistuba II Haymaker C Solis Soto LM. Pathological response and immune biomarker assessment in non-small-cell lung carcinoma receiving neoadjuvant immune checkpoint inhibitors. Cancers. (2022) 14:2775. doi: 10.3390/cancers14112775 35681755 PMC9179283

[20] Berben L Wildiers H Marcelis L Antoranz A Bosisio F Hatse S. Computerised scoring protocol for identification and quantification of different immune cell populations in breast tumour regions by the use of qupath software. Histopathology. (2020) 77:79–91. doi: 10.1111/his.14108 32281132

[21] Jiang H Yu X Li N Kong M Ma Z Zhou D. Efficacy and safety of neoadjuvant sintilimab, oxaliplatin and capecitabine in patients with locally advanced, resectable gastric or gastroesophageal junction adenocarcinoma: early results of a phase 2 study. J Immunotherapy Cancer. (2022) 10:e003635. doi: 10.1136/jitc-2021-003635 PMC8928365 35296556

[22] Lin J-L Lin J-X Lin JP Zheng C-H Li P Xie J-W. Safety and efficacy of camrelizumab in combination with nab-paclitaxel plus S-1 for the treatment of gastric cancer with serosal invasion. Front Immunol. (2021) 12:783243. doi: 10.3389/fimmu.2021.783243 35116023 PMC8805791

[23] Guo H Pa D Sun C Yang P Tian Y Liu Y. Efficacy and safety of sintilimab plus xelox as a neoadjuvant regimen in patients with locally advanced gastric cancer: A single-arm, open-label, phase ii trial. Front Oncol. (2022) 12:927781. doi: 10.3389/fonc.2022.927781 36091139 PMC9458882

[24] Li Z Shan F Wang Y Zhang Y Zhang L Li S. Correlation of pathological complete response with survival after neoadjuvant chemotherapy in gastric or gastroesophageal junction cancer treated with radical surgery: A meta-analysis. PLoS ONE (2018). 13(1):e0189294. doi: 10.1371/journal.pone.0189294 29370182 PMC5784899

[25] Yuan S-Q Nie R-C Jin Y Liang C-C Li Y-F Jian R. Perioperative toripalimab and chemotherapy in locally advanced gastric or gastro-esophageal junction cancer: A randomized phase 2 trial. Nat Med. (2024) 30:552–9. doi: 10.1038/s41591-023-02721-w 38167937

[26] Moehler MH Kato K Arkenau H-T Oh D-Y Tabernero J Cruz-Correa M. Rationale 305: phase 3 study of tislelizumab plus chemotherapy vs placebo plus chemotherapy as first-line treatment (1l) of advanced gastric or gastroesophageal junction adenocarcinoma (Gc/gejc). J Clin Oncol. (2023) 41(4_suppl):286. doi: 10.1200/JCO.2023.41.4_suppl.286

[27] Forde PM Spicer J Lu S Provencio M Mitsudomi T Awad MM. Neoadjuvant nivolumab plus chemotherapy in resectable lung cancer. New Engl J Med. (2022) 386:1973–85. doi: 10.1056/NEJMoa2202170 PMC9844511 35403841

[28] Rothschild SI Zippelius A Eboulet EI Savic Prince S Betticher D Bettini A. Sakk 16/14: durvalumab in addition to neoadjuvant chemotherapy in patients with stage iiia(N2) non-small-cell lung cancer-a multicenter single-arm phase ii trial. J Clin Oncology: Off J Am Soc Clin Oncol. (2021) 39:2872–80. doi: 10.1200/JCO.21.00276 34251873

[29] Shu CA Gainor JF Awad MM Chiuzan C Grigg CM Pabani A. Neoadjuvant atezolizumab and chemotherapy in patients with resectable non-small-cell lung cancer: an open-label, multicentre, single-arm, phase 2 trial. Lancet Oncol. (2020) 21:786–95. doi: 10.1016/S1470-2045(20)30140-6 32386568

[30] Zhao Z-R Yang C-P Chen S Yu H Lin Y-B Lin Y-B. Phase 2 trial of neoadjuvant toripalimab with chemotherapy for resectable stage iii non-small-cell lung cancer. Oncoimmunology. (2021) 10:1996000. doi: 10.1080/2162402X.2021.1996000 34712513 PMC8547836

[31] Apetoh L Ladoire S Coukos G Ghiringhelli F. Combining immunotherapy and anticancer agents: the right path to achieve cancer cure? Ann Oncology: Off J Eur Soc Med Oncol. (2015) 26:1813–23. doi: 10.1093/annonc/mdv209 25922066

[32] Peng J Hamanishi J Matsumura N Abiko K Murat K Baba T. Chemotherapy induces programmed cell death-ligand 1 overexpression via the nuclear factor-κb to foster an immunosuppressive tumor microenvironment in ovarian cancer. Cancer Res. (2015) 75:5034–45. doi: 10.1158/0008-5472.CAN-14-3098 26573793

[33] Bankhead P Loughrey MB Fernández JA Dombrowski Y McArt DG Dunne PD. Qupath: open source software for digital pathology image analysis. Sci Rep. (2017) 7:16878. doi: 10.1038/s41598-017-17204-5 29203879 PMC5715110

[34] Tang X Li M Wu X Guo T Zhang L Tang L. Neoadjuvant pd-1 blockade plus chemotherapy induces a high pathological complete response rate and anti-tumor immune subsets in clinical stage iii gastric cancer. Oncoimmunology. (2022) 11:2135819. doi: 10.1080/2162402X.2022.2135819 36268179 PMC9578498

[35] Yang G Su X Yang H Luo G Gao C Zheng Y. Neoadjuvant programmed death-1 blockade plus chemotherapy in locally advanced esophageal squamous cell carcinoma. Ann Trans Med. (2021) 9:1254. doi: 10.21037/atm-21-3352 PMC8421958 34532391

[36] Thaker PH Bradley WH Leath CA Gunderson Jackson C Borys N Anwer K. Gen-1 in combination with neoadjuvant chemotherapy for patients with advanced epithelial ovarian cancer: A phase I dose-escalation study. Clin Cancer Research: Off J Am Assoc Cancer Res. (2021) 27:5536–45. doi: 10.1158/1078-0432.CCR-21-0360 PMC9338778 34326131

[37] Cascone T William WN Weissferdt A Leung CH Lin HY Pataer A. Neoadjuvant nivolumab or nivolumab plus ipilimumab in operable non-small cell lung cancer: the phase 2 randomized neostar trial. Nat Med. (2021) 27:504–14. doi: 10.1038/s41591-020-01224-2 PMC8818318 33603241

[38] Kim K-J Lee KS Cho HJ Kim YH Yang HK Kim WH. Prognostic implications of tumor-infiltrating foxp3+ Regulatory T cells and cd8+ Cytotoxic T cells in microsatellite-unstable gastric cancers. Hum Pathol. (2014) 45:285–93. doi: 10.1016/j.humpath.2013.09.004 24331841

[39] Iwai Y Ishida M Tanaka Y Okazaki T Honjo T Minato N. Involvement of pd-L1 on tumor cells in the escape from host immune system and tumor immunotherapy by pd-L1 blockade. Proc Natl Acad Sci. (2002) 99:12293–7. doi: 10.1073/pnas.192461099 PMC129438 12218188

[40] Farhood B Najafi M Mortezaee K. Cd8 + Cytotoxic T lymphocytes in cancer immunotherapy: A review. J Cell Physiol. (2019) 234:8509–21. doi: 10.1002/jcp.27782 30520029

[41] Grisaru-Tal S Itan M Klion AD Munitz A. A new dawn for eosinophils in the tumour microenvironment. Nat Rev Cancer. (2020) 20:594–607. doi: 10.1038/s41568-020-0283-9 32678342

[42] Borst J Ahrends T Bąbała N Melief CJM Kastenmüller W. Cd4+ T cell help in cancer immunology and immunotherapy. Nat Rev Immunol. (2018) 18:635–47. doi: 10.1038/s41577-018-0044-0 30057419

[43] Takeuchi A Saito T. Cd4 ctl, a cytotoxic subset of cd4+ T cells, their differentiation and function. Front Immunol. (2017) 8:194. doi: 10.3389/fimmu.2017.00194 28280496 PMC5321676

[44] Bourgeois C Rocha B Tanchot C. A role for cd40 expression on cd8 + T cells in the generation of cd8 + T cell memory. Science. (2002) 297:2060–3. doi: 10.1126/science.1072615 12242444

[45] Engelhard V Conejo-Garcia JR Ahmed R Nelson BH Willard-Gallo K Bruno TC. B cells and cancer. Cancer Cell. (2021) 39:1293–6. doi: 10.1016/j.ccell.2021.09.007 34597591

[46] Wang S-S Liu W Ly D Xu H Qu L Zhang L. Tumor-infiltrating B cells: their role and application in anti-tumor immunity in lung cancer. Cell Mol Immunol. (2019) 16:6–18. doi: 10.1038/s41423-018-0027-x 29628498 PMC6318290

[47] Habif G Crinier A André P Vivier E Narni-Mancinelli E. Targeting natural killer cells in solid tumors. Cell Mol Immunol. (2019) 16:415–22. doi: 10.1038/s41423-019-0224-2 PMC6474204 30911118

[48] Voskoboinik I Smyth MJ Trapani JA. Perforin-mediated target-cell death and immune homeostasis. Nat Rev Immunol. (2006) 6:940–52. doi: 10.1038/nri1983 17124515

[49] Tang X. Tumor-associated macrophages as potential diagnostic and prognostic biomarkers in breast cancer. Cancer Lett. (2013) 332:3–10. doi: 10.1016/j.canlet.2013.01.024 23348699

[50] Murray PJ Allen JE Biswas SK Fisher EA Gilroy DW Goerdt S. Macrophage activation and polarization: nomenclature and experimental guidelines. Immunity. (2014) 41:14–20. doi: 10.1016/j.immuni.2014.06.008 25035950 PMC4123412

[51] Wei J Lu X Liu Q Li L Liu S Liu F. Case report: neoadjuvant pd-1 blockade plus concurrent chemoradiotherapy in unresectable locally advanced gastric cancer patients. Front Oncol. (2021) 10:554040. doi: 10.3389/fonc.2020.554040 33634011 PMC7901487

[52] Manji GA Lee S Del Portillo A May M Ana SS Alouani E. Chemotherapy and immune checkpoint blockade for gastric and gastroesophageal junction adenocarcinoma. JAMA Oncol. (2023) 9:1702–7. doi: 10.1001/jamaoncol.2023.4423 PMC10587824 37856106

[53] Xing X Shi J Jia Y Dou Y Li Z Dong B. Effect of neoadjuvant chemotherapy on the immune microenvironment in gastric cancer as determined by multiplex immunofluorescence and T cell receptor repertoire analysis. J Immunotherapy Cancer. (2022) 10:e003984. doi: 10.1136/jitc-2021-003984 PMC8971786 35361730

