# Supplementary material for: Immune microenvironment features underlying the superior efficacy of neoadjuvant immunochemotherapy over chemotherapy in local advanced gastric cancer
Source: Front Immunol. 2025 Jan 27;16:1497004. doi: 10.3389/fimmu.2025.1497004 (PMC11808021; doi:10.3389/fimmu.2025.1497004)
Supplement: Supplementary file 1 [file DataSheet1.pdf]

Figure S1

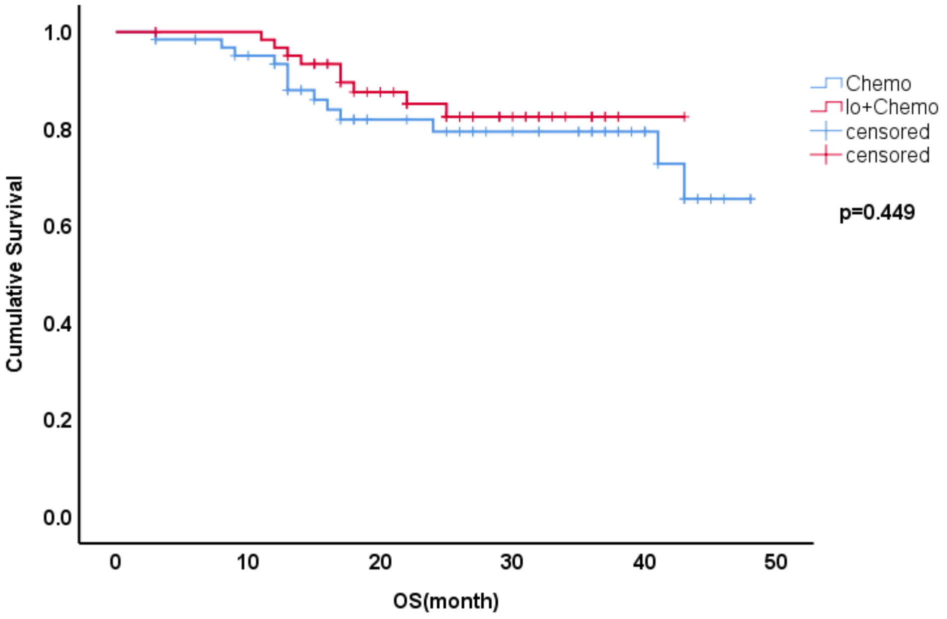

Figure S1. Comparison of overall survival in patients treated with neoadjuvant Io+Chemo versus Chemo; p=0.449.
