# Supplementary material for: Immune microenvironment features underlying the superior efficacy of neoadjuvant immunochemotherapy over chemotherapy in local advanced gastric cancer
Source: Front Immunol. 2025 Jan 27;16:1497004. doi: 10.3389/fimmu.2025.1497004 (PMC11808021; doi:10.3389/fimmu.2025.1497004)
Supplement: Supplementary file 2 [file DataSheet2.pdf]

Figure S2

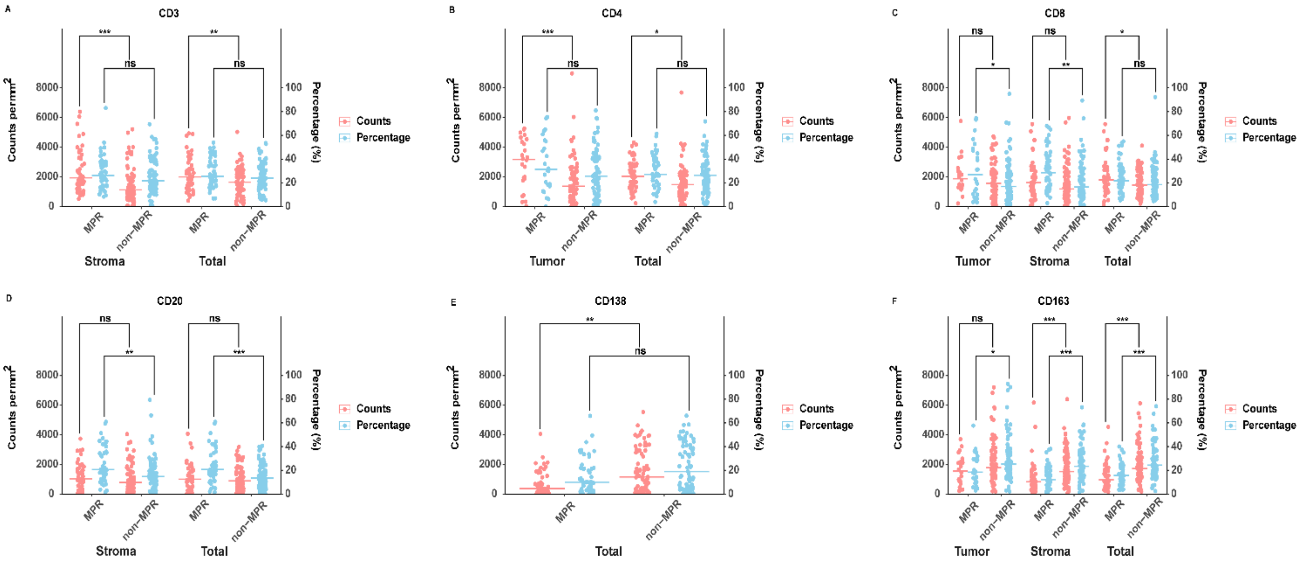

Figure S2. The association between TIICs and pathologic response. The scatter plot was shown as median. The density and percentage of CD3**(A)**, CD4**(B)**, CD8**(C)**, CD20**(D)**, CD138**(E)**, CD163**(F)** were statistical different between MPR and non-MPR. Figure was created with R. MPR, major pathological response; *p<0.05; **p<0.01; ***p<0.001; ns, no statistical significance.
