# Supplementary material for: Immune microenvironment features underlying the superior efficacy of neoadjuvant immunochemotherapy over chemotherapy in local advanced gastric cancer
Source: Front Immunol. 2025 Jan 27;16:1497004. doi: 10.3389/fimmu.2025.1497004 (PMC11808021; doi:10.3389/fimmu.2025.1497004)
Supplement: Supplementary file 3 [file DataSheet3.pdf]

Table S1 Comparison of TIIC subsets in patients treated with neoadjuvant Io+Chemo versus Chemo

	NAT (n=128)														
	Counts/mm^2					Percentage					H-score				
	Io+Chemo		Chemo		P-value	Io+Chemo		Chemo		P-value	Io+Chemo		Chemo		P-value
	Median	Min~Max	Median	Min~Max		Median	Min~Max	Median	Min~Max		Median	Min~Max	Median	Min~Max	
CD3															
Tumor (n=42 vs 59)	2374.4	91.2~6386.4	1058	3.2-5081	**0.000**	24.93	0.45~92.114	19.83	0.07~63.024	0.546	40.077	6.626~150.446	38.49	2.13~150.446	0.675
Stroma (n=63 vs 65)	1794.4	28.80~6064	1241.6	12.8-6391.2	0.128	25.17	0.62~56.77	22.872	0.51~83	0.939	38.79	0.62~95.43	34.672	0.51~139.308	0.527
Total (n= 42 vs 59)	1434.8	379.2~4890.4	1458.4	46.265-5030	**0.000**	25.36	5.62~53.90	23.97	5.36~52.77	0.764	28.78	4.62~99.76	40.089	16.801~96.755	0.223
CD4															
Tumor (n=42 vs 59)	2200.8	30.67~8877.6	1361.6	9.6~5195.2	**0.022**	34.515	0.19~75.41	31.06	0~80.98	**0.020**	56.261	0.21~109.172	29.65	0.04~131.81	**0.010**
Stroma (n=63 vs 65)	1648	28.80~6773.6	1298.4	16.8~3587.2	**0.006**	24.82	0.86~70.36	20.49	0.68~62.478	0.190	34.9	0.86~123.36	31.898	0.74~113.68	0.536
Total (n= 42 vs 59)	1904.8	205.6~7688.4	1530	74.8~3648.4	**0.010**	28.597	1.68~61.34	25.71	0.953~71.729	0.055	43.003	2.07~91.02	34.396	0.962~122.745	0.087
CD8															
Tumor (n=42 vs 59)	2259.6	16~4203.2	1288.10	13.60~5766.6	**0.017**	15.02	0.15~94.95	18.744	0.57~70.756	0.772	31.47	2.25~98.816	12.73	0.17~996.8	**0.016**
Stroma (n=63 vs 65)	1772	82.4~5974.4	975.2	7.2~5671.2	**0.000**	26.24	5.72~89.4	17.423	0.29~56.24	**0.001**	23.53	0~117.32	24.57	0.29~114.302	0.930
Total (n= 42 vs 59)	1982.935	82.4~5558.4	1124.4	195.6~3950.9	**0.000**	20.345	6.42~92.175	20.82	4.58~44.92	0.670	31.61	0~117.18	27.579	2.338~90.02	0.169
CD20															
Tumor (n=42 vs 59)	1238.4	8~5787.2	296.8	0~3913.6	**0.000**	18.712	0.11~71.568	3.33	0~41.228	**0.000**	25.410	0.16~109.744	4.50	0~51.17	**0.000**
Stroma (n=63 vs 65)	1028	0~3514.40	744.8	0.8~4044	0.189	19.41	0~60.86	16.094	5.48~79.47	0.343	22.92	0~90.81	23.198	0.05~117.13	0.488
Total (n= 42 vs 59)	1115.6	0~4066.80	574	0.4~3184.8	**0.002**	20.12	0.12~60.86	12.433	0.015~39.805	**0.000**	23.761	0~72.53	18.365	0.025~78.101	0.117
CD38															
Tumor (n=42 vs 59)	2021.6	0.38~7823.2	1470.4	5.60~5933.60	0.332	17.22	0.10~72.75	26.262	0.930~97.2	0.349	14.85	0.1~113.73	35.752	0.41~162.104	0.055
Stroma (n=63 vs 65)	1384.8	0.8~6623.2	623.2	0~6928	**0.011**	18.02	0.04~96.5	12.43	0~97.234	0.362	23.78	0.04~114.33	18.540	0~127.118	0.232
Total (n= 42 vs 59)	1580.4	36~5656.8	1128	13.735~4699.	**0.039**	15.85	1.39~80.52	20.51	0.51~96.719	0.308	22.45	1.63~141.33	28.83	0.638~129.756	0.547
CD138															
Tumor (n=42 vs 59)	3349.2	8.8~7998.4	622.4	0~8468	**0.000**	51.411	0.119~92.146	8.42	0~96.562	**0.000**	60.415	0.119~181.324	10.6	0~223.24	**0.000**
Stroma (n=63 vs 65)	295.6	3.2~4966.4	174.4	0~4351.2	0.657	8.02	0.088~52.3	2.34	0~56.976	**0.022**	10.886	0.088~76.63	2.45	0~79.216	**0.032**
Total (n= 42 vs 59)	1237.2	17.2~5534.4	496.4	0~4630.4	**0.002**	21.595	0.25~66.04	7.62	0~58.3	**0.003**	29.602	0.29~96.80	9.38	0~117.194	**0.000**
CD56															
Tumor (n=42 vs 59)	359.6	0~8397.6	36	0~2346.4	**0.002**	8.853	0.148~83.248	1.07	0~41.64	**0.000**	4.543	0~92.903	1.215	0~42.358	**0.001**
Stroma (n=63 vs 65)	197.6	0.74~7768.8	76.8	0~2616.8	**0.007**	3.954	0~73.496	1.15	0~33.15	**0.009**	3.51	0.008~73.532	0.84	0~35.5	**0.000**
Total (n= 42 vs 59)	396.8	0.37~6072.8	159	0~1597.2	**0.000**	6.73	0.195~71.616	2.045	0~21.61	**0.000**	5.54	0.07~72.298	1.245	0~30.i835	**0.000**
CD68															
Tumor (n=42 vs 59)	1117.2	12.8~4952.8	368	0.8~4324	**0.000**	11.384	2.22~80.18	10.3	0.03~36.542	0.074	12.096	0.18~109.4	9.496	0.03~47.648	0.067
Stroma (n=63 vs 65)	264	0.8~2995.2	323.2	4.8~6765	0.970	5.040	0.02~66.26	6.08	0.13~226.08	0.906	5.703	0.02~57.52	6.8	0.13~113.64	0.946
Total (n= 42 vs 59)	470	6.8~3286.53	370.4	15.2~3812.5	0.053	7.541	1.655~55.10	8.08	0.38~120.235	0.228	8.256	0.1~57.52	9.045	0.38~65.36	0.462
CD163															
Tumor (n=42 vs 59)	1374.9	191.2~6825.6	2156.8	165.6~7196.8	**0.001**	20.885	3.09~58.71	29.374	2.178~92.916	**0.003**	23.19	3.36~73.64	42.24	2.402~144.976	**0.000**
Stroma (n=63 vs 65)	1096	44~6172	1271.2	104.8~6396	0.693	19.51	1.17~73.17	19.568	2.57~58.57	0.418	22.69	1.17~125.45	22.72	2.86~84.806	0.501
Total (n= 42 vs 59)	994.4	117.6~4351.6	1706	169.2~6124.8	**0.018**	19.575	3.71~54.245	22.95	2.530~74.06	**0.004**	21.8	3.88~76.07	30.536	2.829~113.848	**0.007**

Table S2 TIIC subsets in MPR versus non-MPR

	NAT(n=128)														
	Counts/mm^2					Percentage					H-score				
	TRG 1-2		TRG 3-5		P-value	TRG 1-2		TRG 3-5		P-value	TRG 1-2		TRG 3-5		P-value
	Median	Min~Max	Median	Min~Max		Median	Min~Max	Median	Min~Max		Median	Min~Max	Median	Min~Max	
CD3															
Tumor (n=24 vs 77)	158.72	2.73~57.746	1628.8	3.2~6386.4	0.781	27.369	2.73~57.746	21.105	0.07~92.114	0.825	48.855	2.13~124.1	36.18	3.89~150.4	**0.044**
Stroma (n=51 vs 77)	1936.8	516.8~6391.2	1117.3	12.8~5211.2	**0.000**	26.08	8.32~83	21.54	0.51~69.406	**0.238**	39.98	9.23~105.3	32.438	0.51~139.3	0.165
Total (n=24 vs 77)	2000.8	394.4~4929.2	1648.4	46.265~5030	**0.007**	25.39	6.80~53.90	23.885	5.36~53.447	0.208	40.93	13.48~99.7	38.929	3.68~96.75	0.432
CD4															
Tumor (n=24 vs 77)	3175	21.4~5264.80	1361.6	9.6~8977.60	**0.001**	31.208	0~75.41	25.6	0.04~80.98	0.150	38.044	5.13~523.4	33.436	0.04~131.8	0.468
Stroma (n=51 vs 77)	1726.4	421~4080.4	1143.2	16.8~6773.60	0.091	20.06	7.74~59.235	22.216	0.68~70.36	0.117	39	8.3~94.752	30.42	0.74~123.3	0.264
Total (n=24 vs 77)	2037.6	221.2~4319.2	1472.4	74.8~7688.4	**0.012**	27.15	3.87~61.34	26.356	0.953~71.729	0.204	41.12	7.165~87.6	38.377	0.962~122.	0.327
CD8															
Tumor (n=24 vs 77)	1880.8	208~5766.67	1564.8	13.6~4732	0.289	27.022	3.35~74.45	16.942	0.15~94.95	**0.044**	21.63	3.75~74.56	27.046	0.17~98.81	0.382
Stroma (n=51 vs 77)	1606.4	82.4~5558.4	1175.2	7.2~5974.4	0.179	28.56	2.865~67.96	16.32	0.29~89.4	**0.002**	21.186	0~117.18	22.11	0.22~117.3	0.514
Total (n=24 vs 77)	1791.2	82.4~5558.4	1456	195.6~4101.6	**0.025**	21.79	5.217~54.895	18.421	4.58~92.175	0.234	29.17	0~117.18	28.725	1.24~98.11	0.512
CD20															
Tumor (n=24 vs 77)	708.4	12~5787.2	511.2	0~4348.8	0.284	8.965	0.1~61.006	10.14	0~71.568	0.891	12.4	0.13;91.742	10.42	0~109.744	0.879
Stroma (n=51 vs 77)	1032	0~3733.6	782.4	0~4044	0.410	20.66	1.48~60.86	14.8	0~79.47	**0.003**	28.45	0~81.354	20.713	0~117.13	0.480
Total (n=24 vs 77)	998.8	0~4066.8	888	0.4~3184.8	0.593	20.69	1.47~60.86	13.592	0.015~40.69	**0.000**	24.732	0~78.101	19.29	0.025~71.3	0.261
CD38															
Tumor (n=24 vs 77)	2235.45	218.67~7823.2	1393.6	0.38~5399.6	0.059	22.18	1.34~96.203	19.33	0.1~72.5	0.664	31.04	1.88~162.1	26.386	0.1~107.99	0.210
Stroma (n=51 vs 77)	1228.8	36~6928	595.2	0~6623.2	0.229	18.02	1.39~97.234	10.375	0~96.5	0.497	25.79	1.63~141.3	13.97	0~136.4	0.124
Total (n=24 vs 77)	1442	36~5656.8	1173.6	13.735~4969.2	0.194	21.055	1.39~91.719	19.135	0.51~84.3	0.482	27.449	1.63~141.3	25.327	0.638~122.	0.145
CD138															
Tumor (n=24 vs 77)	540.5	29.6~7111.2	1790.4	0~8468	0.061	22.314	0.64~92.146	33.178	0~96.502	0.269	18.196	0.91~148.5	36.364	0~223.24	0.179
Stroma (n=51 vs 77)	238.8	0~3855.2	215.2	0~4966.4	0.780	7.3	0~49.43	4.032	0~56.976	0.254	10.93	0~76.63	4.389	0~79.216	0.064
Total (n=24 vs 77)	378.8	0~4160.6	1157.2	0~5534.4	**0.007**	10.03	0~65.87	18.945	0.005~66.04	0.080	12.181	0~88.52	19.658	0.005~117.	0.146
CD56															
Tumor (n=24 vs 77)	161.6	1.6~6433.333	200	0~8397.6	0.325	5.05	8.03~71.703	3.74	0~83.248	0.172	2.935	0.03~92.90	2.976	0~90.044	0.247
Stroma (n=51 vs 77)	290	0~2616.8	62.4	0~7768.8	0.171	5.54	0~49.11	1.15	0~73.496	0.058	2.672	0~46.327	0.85	0~73.532	0.068
Total (n=24 vs 77)	396.8	0~3266.667	161.6	0~6072.8	0.243	6.31	0~49.11	2.560	0~71.616	0.074	4.105	0~66.411	2.2	0~72.298	0.137
CD68															
Tumor (n=24 vs 77)	969.2	97.33~4458.667	540.4	0.8~4952.8	0.121	11.89	1.32~52.16	10.41	0.03~80.18	0.879	13.74	1.32~65.27	10.55	0.03~109.1	0.593
Stroma (n=51 vs 77)	306.4	15.2~2995.2	272.8	0.8~67.65	0.125	6.45	0.38~66.26	4.59	0.02~86.08	0.487	6.966	0.38~57.52	5.703	0.02~113.6	0.311
Total (n=24 vs 77)	388.8	15.2~3286.53	410.8	6.8~3812.5	0.371	7.357	0.38~55.10	8.025	1.15~50.235	0.909	8.375	0.38~57.52	8.279	0.1~65.36	0.989
CD163															
Tumor (n=24 vs 77)	1563.6	2.4~3704.8	1778.4	165.6~7196.8	0.060	18.435	3.08~57.716	25.266	2.179~92.916	**0.035**	24.51	3.5~138.7	30.95	2.402~144.	0.388
Stroma (n=51 vs 77)	824.8	104.8~6172	1522.4	44~6396	**0.000**	12.26	1.37~38.11	23.38	1.17~73.17	**0.000**	15.391	1.5~72.38	24.99	1.17~125.4	**0.001**
Total (n=24 vs 77)	972	164~4531.6	1716.8	169.2~6124.8	**0.000**	15.59	3.71~40.051	24.34	2.53~74.06	**0.000**	19.725	3.88~100.1	29.975	2.829~113.	**0.003**

Table S3 TIIC subsets in MPR versus non-MPR patients in neoadjuvant Io+Chemo group

	NAT(n=63)														
	Counts/mm^2					Percentage					H-score				
	TRG1-2		TRG3-5		P-value	TRG1-2		TRG3-5		P-value	TRG1-2		TRG3-5		P-value
	Median	Min~Max	Median	Min~Max		Median	Min~Max	Median	Min~Max		Median	Min~Max	Median	Min~Max	
CD3															
Tumor (n=10 vs 32)	2308	316.8~4227.2	2374.4	91.2~6386.4	0.969	40.29	5.28~57.746	20.59	0.45~92.114	0.038	46.87	16.63~61.9	40.521	6.624~150.	**0.607**
Stroma (n=31 vs 32)	2118.8	758.4~6064	1283.6	28.8~3673.6	**0.001**	24.18	8.32~53.90	25.935	0.62~56.77	**0.730**	34.2	9.23~95.43	39.035	0.62~76.08	0.321
Total (n=10 vs 32)	2262.4	650~4929.2	2036.4	167.6~3558	**0.053**	25.815	6.8~53.90	22.784	5.62~53.447	0.177	34.2	13.48~99.7	41.589	3.68~96.40	0.999
CD4															
Tumor (n=10 vs 32)	3225.8	1757.33~5264.8	1776	30.67~8977.6	**0.078**	30.95	17.13~75.41	39.526	0.19~66.498	0.968	66.759	10~92.13	59.261	0.21~109.1	0.883
Stroma (n=31 vs 32)	1698.8	631.2~4080.4	1634.4	28.8~6773.6	0.922	24.78	8.73~47.26	26.565	0,86~70.36	0.912	34.08	9.49~72.52	35.88	0.86~123.3	0.869
Total (n=10 vs 32)	1938	631.2~4319.2	1610.8	205.6~7688.4	**0.883**	26.06	8.73~61.34	30.037	1.68~59.47	0.291	40.26	9.49~87.69	45.565	2.07~91.02	0.220
CD8															
Tumor (n=10 vs 32)	2357.4	1138.4~3694	2047.6	16~4203.2	0.357	49.92	10.5~74.45	10.91	0.15~94.95	**0.001**	26.035	7.675~64.3	33.82	2.25~98.81	0.417
Stroma (n=31 vs 32)	1808	82.4~5558.4	1692.8	502.4~5974.4	0.773	35.08	11.6~67.96	18.53	5.72~89.4	**0.034**	24.8	0~117.18	22.82	0.22~117.3	0.763
Total (n=10 vs 32)	1982.935	82.4~5558.4	1966.4	687.6~4101.6	**0.536**	21.79	6.42~54.895	15.913	6.535~92.175	0.427	26.035	0~117.18	41.015	1.24~98.11	0.620
CD20															
Tumor (n=10 vs 32)	1645.6	140.8~5787.2	1206	8~4348.8	0.145	17.153	1.44~61.006	19.052	0.11~71.568	0.814	22.565	0.74~91.74	27.749	0.16~109.7	0.778
Stroma (n=31 vs 32)	1028	0~2985.6	1025.6	0~3514.4	0.991	20.66	3.04~60.86	17.285	0~33.70	**0.032**	20.2	0~72.53	23.5	0~90.81	0.177
Total (n=10 vs 32)	1032	0~4066.8	1174.2	57.2~2918.8	0.564	22.17	4.88~60.86	18.21	0.12~40.69	**0.099**	22.055	0~72.53	28.329	1.09~71.38	0.117
CD38															
Tumor (n=10 vs 32)	2304	360.8~7823.8	1734.8	0.38~4506.4	0.262	25.595	6.52~72.75	6.93	0.1~65.53	0.052	31.04	3.36~113.7	8.71	0.1~107.99	0.056
Stroma (n=31 vs 32)	1442	36~5656.8	1081.2	0.8~6623.2	0.821	19.49	1.39~80.52	10.73	0.04~96.5	0.339	25.88	1.63~141.3	13.92	0.04~136.4	0.992
Total (n=10 vs 32)	1580.4	36~5656.8	1667.8	120~4969.2	0.943	21.44	1.39~80.52	14.44	2.01~69.59	0.289	29.56	1.63~141.3	20.205	2.18~122.2	0.387
CD138															
Tumor (n=10 vs 32)	1287	42.4~7111.2	4849.6	8.8~7998.4	0.043	44.260	17.24~92.146	58.919	0.119~90.914	0.669	36.235	1.612~148.	80.745	0.119~181.	0.219
Stroma (n=31 vs 32)	295.6	3.2~2488	306.4	6.4~4966.4	0.631	10.1	0.57~49.43	6.178	0.088~52.3	0.127	18.77	0.58~76.63	7.102	0.088~62.1	0.011
Total (n=10 vs 32)	543.9	36.8~4061.6	2836.2	17.2~5534.4	**0.000**	12.75	1.3~65.87	34.198	0.25~66.04	0.023	20.51	0.58~88.52	48.112	0.29~96.8	0.021
CD56															
Tumor (n=10 vs 32)	923.2	24~6433.333	328.8	0~8397.6	0.385	25.43	0.959~71.703	5.291	0.148~83.248	0.104	5.425	0.67~92.90	4.543	0~90.044	0.408
Stroma (n=31 vs 32)	340	22.5~2604.8	66	0.74~7768.8	0.953	6.28	0~49.11	2.831	0.008~73.496	0.422	5.54	0.36~46.32	1.261	0.088~73.5	0.347
Total (n=10 vs 32)	509.6	22.5~3266.667	206.2	0.37~6072.8	0.998	10.785	0.29~49.11	5.744	0.195~71.616	0.396	6.435	0.36~66.41	2.988	0.07~72.29	0.627
CD68															
Tumor (n=10 vs 32)	2138.8	832.8~4458.667	604	12.8~4952.8	0.079	19.583	2.22~52.16	10.701	2.939~80.18	0.982	22.335	4.51~65.27	11.237	0.18~109.1	0.507
Stroma (n=31 vs 32)	428.8	86.4~2995.2	202	0.8~1565.6	0.002	9.1	1.655~66.26	3.708	0.02~24.092	0.002	8.357	1.742~57.5	4.294	0.02~29.32	0.006
Total (n=10 vs 32)	428.8	86.4~3286.53	481.4	6.8~2824.8	0.345	9.1	1.655~55.10	7.445	2.05~40.10	0.585	8.357	1.742~57.5	8.225	0.1~56.58	0.715
CD163															
Tumor (n=10 vs 32)	1093.65	236.8~2194.4	1437.6	191.2~6825.6	0.225	13.94	4.77~29.46	22.47	3.09~58.71	**0.041**	19.46	5.15~49.95	24.285	3.36~73.64	0.423
Stroma (n=31 vs 32)	846.4	128~6172	1390.4	44~4447.2	**0.144**	11.46	1.37~37.4	23.445	1.17~73.17	**0.001**	13.48	1.50~72.38	24.065	1.17~125.4	**0.090**
Total (n=10 vs 32)	825.6	164~4531.60	1381	287.6~5450.8	**0.060**	11.46	1.37~37.4	24.005	5.095~54.245	**0.000**	13.48	3.88~72.38	27.015	4.49~76.07	**0.126**

Table S4 TIIC subsets in MPR versus non-MPR patients in neoadjuvant Chemo group

	NAT(n=65)														
	Counts/mm^2					Percentage					H-score				
	TRG1-2		TRG3-5		P-value	TRG1-2		TRG3-5		P-value	TRG1-2		TRG3-5		P-value
	Median	Min~Max	Median	Min~Max		Median	Min~Max	Median	Min~Max		Median	Min~Max	Median	Min~Max	
CD3															
Tumor (n=14 vs 45)	1154.8	272~2908	1058	3.2~5081.6	0.707	13.48	2.73~56.78	24.572	0.07~63.042	0.11	53.45	2.13~124.17	35.198	3.89~150.44	0.137
Stroma (n=20 vs 45)	1730	516.8~6391.	1017.6	12.8~5211.2	0.153	28.415	9.303~83	19.676	0.51~69.406	0.189	44.69	14.146~105.	29.3688	0.51~139.30	0.226
Total (n=14 vs 45)	1552.8	394.4~3515.	1213.6	46.265~503	0.242	23.555	6.91~49.116	23.967	5.36~52.77	0.771	52.605	21.055~89.7	33.929	16.801~96.7	**0.019**
CD4															
Tumor (n=14 vs 45)	2607.6	21.47~5195.	1249.6	9.6~3792	0.075	35.587	0~74.306	20.65	0.04~80.98	0.078	36.274	5.13~123.43	27.058	0.04~231.81	0.298
Stroma (n=20 vs 45)	1876.8	421~3461.6	1004	16.8~3587.2	**0.017**	29.162	7.74~59.235	19.600	0.68~62.478	0.099	44.372	8.3~94.752	27.117	0.74~113.68	0.080
Total (n=14 vs 45)	2246.6	221.2~3648.	1329.2	74.8~2738	**0.002**	34.657	3.87~59.235	22.018	0.953~71.72	**0.018**	45.285	7.165~85.93	32.051	0.962~122.7	**0.037**
CD8															
Tumor (n=14 vs 45)	1393.6	208~5766.6	1096	13.6~4732	0.507	16.116	3.15~48.726	18.744	0.57~70.756	0.411	9.343	3.75~74.562	13.914	0.17~96.8	0.640
Stroma (n=20 vs 45)	1257.2	162.4~3905.	772	7.2~5671.2	0.413	18.283	2.865~44.92	15.024	0.29~56.24	0.230	28.131	3.604~90.02	20.562	0.29~114.30	0.197
Total (n=14 vs 45)	1470.4	308.4~3950.	1100.4	195.6~3042.	0.183	21.455	5.217~44.92	20.82	4.58~40.616	0.567	31.759	6.695~90.02	25.105	2.338~89.41	0.202
CD20															
Tumor (n=14 vs 45)	319.2	12~2688.8	270.4	0~3913.6	0.986	3.215	0.11~26.812	3.33	0~41.228	0.931	4.02	0.13~51.17	5.42	0~50.48	0.877
Stroma (n=20 vs 45)	1157.2	59.2~3733.6	716.8	0.8~4044	0.391	20.485	1.48~47.164	12.509	0.03~79.47	0.195	38.871	1.85~81.354	18.359	0.05~117.13	**0.022**
Total (n=14 vs 45)	927.2	89.6~2053.6	480	0.4~3184.8	0.488	18.763	1.47~34.759	9.622	0.015~39.80	0.067	33.851	1.83~78.101	16.289	0.025~54.99	**0.022**
CD38															
Tumor (n=14 vs 45)	2207.45	218.67~469	1172.8	5.6~5933.6	0.357	20.847	1.34~96.203	26.36	0.930~83.5	0.611	72.387	1.88~162.10	39.792	0.41~98.96	0.986
Stroma (n=20 vs 45)	809.4	148.8~6926	470.4	0~4719.2	**0.023**	16.867	2.23~96.234	10.375	0~83.4	0.599	24.63	2.69~217.22	14.121	0~109.03	**0.030**
Total (n=14 vs 45)	1288.525	202.535~42	1086.1	13.735~469	0.109	16.610	5.761~96.71	20.770	0.51~74.3	0.543	26.139	8.249~129.7	29.598	0.638~82.11	0.541
CD138															
Tumor (n=14 vs 45)	397.6	29.60~2814.	601.6	0~8468	0.293	5.297	0.64~72.766	12.43	0~96.502	0.236	6.173	0.91~78.44	10.906	0~233.24	0.266
Stroma (n=20 vs 45)	179.2	0~3855.20	154.4	0~4351.2	0.972	2.58	0~21.46	2.17	0~56.976	0.458	3.02	0~54.53	2.25	0~79.216	0.594
Total (n=14 vs 45)	315.25	0~2133.6	753.2	0.4~4630.4	0.182	3.173	0~37.103	10.845	0.005~58.3	0.071	8,223	0~39.846	11.195	0.005~117.1	0.261
CD56															
Tumor (n=14 vs 45)	30	1.6~2346.4	52	0~1344	0.505	1.214	0.03~23.56	0.84	0~41.64	0.934	1.242	0.03~42.358	1.04	0~26.17	0.372
Stroma (n=20 vs 45)	281.6	0~2616.8	62.4	0~1959.2	**0.009**	4.19	0~31.25	0.83	0~33.15	0.127	1.25	0~22.97	0.8	0~35.5	0.397
Total (n=14 vs 45)	215.9	0~1352.4	83.6	0~1597.2	0.083	3.958	0~21.61	1.905	0~21.325	0.370	1.223	0.015~25.22	1.705	0~30.835	0.609
CD68															
Tumor (n=14 vs 45)	356.665	97.33~1624	373.6	0.81~4324	0.763	9.515	1.32~31.39	10.4	0.03~36.542	0.752	8.875	1.32~31.06	10.065	0.03~47.648	0.914
Stroma (n=20 vs 45)	224.6	15.2~2188.8	420	4.8~6765	0.593	3.82	0.38~32.69	6.42	0.13~226.08	0.406	4.345	0.38~25.78	7.5	0.13~113.64	0.491
Total (n=14 vs 45)	317.733	15.2~1484.4	393.6	57.2~3812.5	0.529	5.18	0.38~22.495	8.245	1.15~120.23	0.315	7.818	0.38~19.585	9.41	1.165~65.36	0.259
CD163															
Tumor (n=14 vs 45)	2105.6	204~3704.8	2295.2	165.6~7196.	0.124	28.522	3.08~57.716	30	2.179~92.91	0.136	**37.41**	3.5~138.7	43	2.404~144.9	0.500
Stroma (n=20 vs 45)	766.8	104.8~1903.	1594.4	172.8~6396	**0.000**	13.704	2.57~38.11	23.17	2.881~58.57	**0.002**	**16.773**	2.86~61.538	28.284	2.984~84.80	**0.012**
Total (n=14 vs 45)	1323.2	231.6~2464.	1885.6	169.2~6124.	**0.001**	17.953	3.714~40.05	26.795	2.530~74.06	**0.003**	23.49	4.43~100.11	34.115	2.829~113.8	**0.038**
